# REVISION TOTAL KNEE ARTHROPLASTY USING THE MODERN CONSTRAINED CONDYLAR KNEE PROSTHESIS

**DOI:** 10.1590/1413-785220162406146213

**Published:** 2016

**Authors:** Naoki Nakano, Tomoyuki Matsumoto, Hirotsugu Muratsu, Kazunari Ishida, Ryosuke Kuroda, Masahiro Kurosaka

**Affiliations:** 1. Kobe University, Graduate School of Medicine, Department of Orthopaedic Surgery, Chuo-ku, Kobe, Japan.; 2. Steel Memorial Hirohata Hospital, Department of Orthopaedic Surgery, Hirohata-ku, Himeji, Japan.; 3. Kobe Kaisei Hospital, Department of Orthopaedic Surgery, Nada-ku, Kobe, Japan.

**Keywords:** Arthroplasty, replacement, Knee. Knee prosthesis Treatment outcome

## Abstract

**Objective:**

To determine whether the second-generation constrained condylar prosthesis provided satisfactory results in revision total knee arthroplasty.

**Methods:**

A series of 41 cases of revision total knee arthroplasty using the second-generation constrained condylar knee prosthesis was reviewed. The series comprised 7 men and 34 women with a mean age of 73.2 years. The original diagnosis was predominantly osteoarthritis. The most common reason for revision surgery was aseptic loosening. The mean interval between the primary and revision surgeries was 66.4 months. The mean follow-up period was 49.4 months.

**Results:**

The mean Knee Society knee score improved from 43.8 to 82.9 after revision surgery, the mean Knee Society function score improved from 37.1 to 79.2; the range of motion improved from 95.6° to 105.6° and the radiological femorotibial alignment improved from 181.4° (varus 6.4°) to 174.9° (valgus 0.1°), on average (*p*<0.001 at all items).

**Conclusion:**

Revision total knee arthroplasty with the use of the second-generation constrained condylar knee prosthesis yielded reproducible clinical success. Level of Evidence IV, Case series.

## INTRODUCTION

The main goal of revision total knee arthroplasty (TKA) is to provide a stable, well-functioning implant in the treatment of the failed arthroplasty. Primary TKA has proven over the past 20 years to be a highly successful surgical procedure, with survivorship rates approaching 95% after a 15-year follow-up period.^1^ However, the results of revision TKA have been less encouraging. The poorer results of revision TKA have been due to multiple factors, including extensor mechanism problems,^2^ malalignment,^3^ and ligamentous instability.^4^

**Figure 1:**
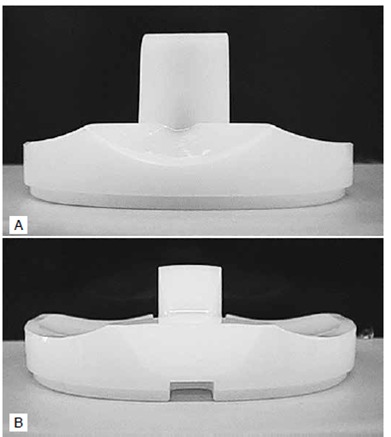
(A) Tibial polyethylene inserts of a constrained condylar prosthesis. Legacy Constrained Condylar Knee prosthesis (LCCK; Zimmer, Warsaw, IN, USA); (B) Posterior stabilized prosthesis (PS). Legacy Knee Posterior Stabilized prosthesis (LPS; Zimmer, Warsaw, IN, USA). An insert of a constrained condylar prosthesis is characterized by the high and broad central post that fits closely against the femoral cam, providing, thus, increased stability.

For revision TKA, in general, a cemented posterior stabilized (PS) prosthesis is used if both collateral ligaments are felt to be competent. In response to the problems caused by ligamentous instability, a constrained condylar knee design that resists the coronal plane moments allowed by deficient soft-tissue constraints was developed. This prosthesis is varus-valgus constrained and unlinked. It features a relatively high and broad central post that fits closely against the femoral cam, providing, thus increased stability. (Figure 1)

If one or both collateral ligaments are completely incompetent or a genu recurvatum can be seen in the knee, a constrained condylar knee design is inadequate to solve the problem. In such a case, a more constrained prosthesis, such as linked-hinge prosthesis, is needed.

The reported clinical outcomes of TKAs performed using constrained condylar knee-type prostheses are generally good. For example, Rosenberg et al.^5^ reported that 35 of 36 knees had excellent or good results after a mean follow-up of 45 months. In addition, Kim et al.^6^ reported no failure in 14 knees after a mean follow-up of 6.3 years. However, one clinical report^7^ found that only about half of 21 knees had good results after a mean duration follow-up of 4 years.

Since 2006, we have been using a second-generation constrained condylar knee prosthesis with a right and left femoral components, modular stem extensions for both the femoral and tibial components, and a new locking mechanism for the constrained tibial polyethylene liner. A feature of the second-generation prosthesis is that it allows 2° of internal-external rotation and 1.25° of varus-valgus angulation, with a redesigned patellofemoral articulation. Theoretically, this design helps the soft tissues around the prosthetic interfaces to absorb force.

The purpose of the present study was to report the clinical scores and overall alignment after revision TKA using this second-generation constrained condylar knee prosthesis.

## MATERIALS AND METHODS

Between 2006 and 2011, 41 revision TKAs in 41 patients using the Legacy Constrained Condylar Knee prosthesis (LCCK; Zimmer, Warsaw, IN, USA) were performed in three hospitals by three experienced surgeons. These constituted the study group. We used this prosthesis for revision surgery when a PS prosthesis was thought to be insufficient because of severe joint instability due to the incompetence of one or both collateral ligaments. In the case of genu recurvatum, we chose linked-hinge prosthesis instead of this constrained condylar (unlinked) prosthesis. In other words, the indication for using this prosthesis was an absent posterior cruciate ligament and a deficient medial or lateral collateral ligament, but an intact quadriceps mechanism. The study was approved by the institutional review board of the participating hospitals, and all patients provided informed consents. The patients' demographic data are summarized in Table 1. These 41 revised total knee arthroplasties were available for review for a minimum of 2 years after revision surgeries. Median follow-up period was 46 months.

**Table 1:** Demographic data on the 41 patients who underwent revision total knee arthroplasty with a Legacy Constrained Condylar Knee prosthesis (LCCK).

Gender (M/F)	7/34
Age (years old) ^a^	57-84 (73.2)
Time between primary and revision surgeries (months) ^a^	1-216 (66.4)
Duration of follow-up (months) ^a^	24-95 (49.4)
Primary diagnosis **^b^**	
Osteoarthritis	35 (85.4)
Rheumatoid arthritis	4 (9.8)
Osteonecrosis of the medial femoral condyle	2 (4.9)
Reason for revision **^b^**	
Aseptic loosening	31 (75.6)
Infection	9 (22.0)
Periprosthetic fracture	1 (2.4)

a Age at the time of the revision surgery. The range is given with the mean in parentheses; ^b^ The number of knees is given with the percentage in parentheses

Prophylactic antibiotics were administered immediately before the skin incision. A longitudinal midline skin incision was used in most cases. All revision prostheses were fixed with cement. A modular stem with one or more (posterior and/or distal) femoral and/or tibial augments was used to achieve joint stability, to fill the bony defect and to achieve balance with the extension/flexion gap. For all the femoral and tibial components, uncemented 145-mm stem extension were used. Tibial metal augments were used in 31 out of 41 knees and femoral metal augments were used in 30 out of 41 knees. Off-set stem was used in 35 out of 41 knees for the tibial component and 34 out of 41 knees for the femoral component. Tibial insert thickness varied from 10mm to 23mm (10 mm, nine knees; 12 mm, five knees; 14 mm, nine knees; 17 mm, nine knees; 20 mm, six knees; and 23 mm, three knees). If there was subluxation or medial lift-off of the patella, a lateral retinacular release was performed from inside out, attempting to preserve the superior lateral geniculate vessels (9 knees).

Nine knees that were infected preoperatively underwent 2-stage revision surgery, which consisted of removal of the previous prosthesis and intravenous antibiotic therapy followed by reimplantation. Infection after primary TKA was diagnosed if any of the following criteria were fulfilled: (1) an abscess or sinus tract communicating with the joint space, (2) positive preoperative aspiration culture findings on solid media, (3) two or more positive intraoperative cultures, or (4) one positive culture on solid media in conjunction with the presence of gross purulence. In patients with negative cultures, infection was diagnosed when any of the following findings were present: elevated white blood cell (WBC) count and leukocyte differential count in the aspirated fluid or an abnormal serology (ESR > 30 mm/h, CRP level > 1.0 mg/dL). In these cases, reimplantation was delayed until all laboratory findings, including WBC count, ESR, CRP level, and analysis of aspirated joint fluid were within normal limits.

All of the clinical and radiological data of the 41 revision TKAs were reviewed by an orthopedic surgeon who had no connection to either the original or the revision surgery, and the results were entered into a computerized record. Routine follow-up evaluation was scheduled at three months, six months, and one year after the revision surgery and yearly thereafter. At these times, the patients were evaluated clinically and radiologically. The preoperative and postoperative review data were summarized according to the scoring systems of the Knee Society.^8^ The clinical results were evaluated on the basis of the range of motion (ROM), Knee Society knee score (KSKS), and Knee Society function score (KSFS). Scores in the ranges of 80 to 100, 70 to 79, 60 to 69, and less than 60 were considered excellent, good, fair, and poor, respectively. For the radiological assessment, standing anteroposterior radiographs, including the femoral head and ankle were evaluated on the basis of the alignment of the limb. If there was any alignment or position change of the components and a radiolucent line was > 1mm in width, a loosening of the component was defined.

All statistical analyses were performed using Microsoft Excel (Microsoft Japan, Inc., Tokyo, Japan). The preoperative and postoperative data were compared using paired Student's *t-*tests. *P*<0.05 was considered to indicate statistical significance.

## RESULTS

The results are shown in Table 2. The clinical results, including the KSKS, KSFS, ROM, and femorotibial angle on anteroposterior radiographs, improved significantly after revision TKA (*p*< 0.001). The KSKS and KSFS indicated excellent or good outcomes in 37 patients (90.2%) and fair outcomes in the remaining four patients. No knee exhibited varus-valgus instability in flexion or extension. The alignments of 11 out of the 41 knees were outside the range of neutral alignment (between 2° varus (+)/valgus (−) from 175°; 175° was considered to be neutral alignment) as defined by the Knee Society (+3° in four knees, −5° in three knees, +5° in two knees, +4° in one knee and −9° in one knee).^8^

**Table 2:** Clinical and radiographic results at the final follow-up examination.

Item	Score *		
	Preoperative	Postoperative	*p* value
Knee Society knee score (points)	43.8 (20-70)	82.9 (50-99)	<0.001
Knee Society function score (points)	37.1 (15-72)	79.2 (70-95)	<0.001
Maximum extension (°)	- 6.8 (25-0)	- 2.1 (10-0)	<0.001
Maximum flexion (°)	102.4 (85-130)	107.7 (85-130)	<0.001
Femorotibial angle (°)^#^	+6.4 (4-+20)	- 0.1 (9-+5)	<0.001

* The mean is given with the range in parentheses; ^#^ 175° is considered to be neutral (0°), + means varus and − means valgus from the neutral; Differences for which *p*<0.05 were considered statistically significant.

The complication rate was 7.3% (three cases of infection). All infections that developed after revision surgery were recurrent infections in knees for which an infection had been the cause of the revision. One of these cases underwent re-revision with an LCCK, whereas the remaining two cases underwent insert exchange only. An asymptomatic calf thrombus was diagnosed during routine ultrasonography in two patients, neither of them had any subsequent complications. No failure due to joint instability, supracondylar femoral fracture, fracture of the tibial post, peroneal nerve palsy, or aseptic loosening of the component assessed by radiographs has occurred.

## DISCUSSION

The most important finding of this study was that patients who undergo revision TKA using the LCCK can achieve statistically significant improvement in clinical scores and overall alignment after surgery with an acceptable complication rate, though surgeons should keep in mind that not everything that is statistically significant is clinically significant. The 90.2% rate of good and excellent results in this study compares favorably with those previously reported for revision TKA.^3^ ^,^ ^5^ The total number of revision TKAs will undoubtedly increase with the advancing age of the population and the increasing number of primary TKAs being performed.^9^ ^,^ ^10^ Although the results of revision TKA are expected to improve as knee surgeons gain more experience with revision total knee procedures and improvements in modern instrumentation systems that facilitate proper anatomic placement of revision prostheses,^11^ we are only halfway to fully satisfactory results. For example, Goldberg et al.^4^ reported good and excellent results in only 46% of revision TKAs performed for mechanical failure after an average follow-up of five years. We, therefore, considered it important to clarify the results of the use of the modern constrained condylar knee prosthesis for revision TKA.

Revision surgery generally requires a more constrained prosthesis than primary TKA, because of the greater extents of ligamentous instability and bony defects. However, surgeons usually prefer to use the least-constrained prosthesis for revision TKA for fear that greater constraint might lead to mechanical loosening and prosthesis failure.^5^ ^,^ ^12^ ^,^ ^13^ However, when a less-constrained prosthesis such as the PS prosthesis is insufficient to achieve the required stability, surgeons are inevitably forced to use a more constrained prosthesis such as the LCCK, which is the most constrained type of unlinked prosthesis.^11^ ^,^ ^14^ The most common indication for the use of such a prosthesis is revision TKA in a patient with soft tissue incompetence.

Haas et al.^15^ reported that infection was the most common reason for failure after revision TKA (3 cases, 4%). Lee et al.^16^ also reported infection to be a major reason for failure after revision (four cases, 5%). However, Kim et al.^17^ reported a 9% complication rate (10 cases) in 114 revision TKAs using LCCKs, with aseptic loosening being the most common cause of failure. Infection, which developed in three of 41 knees, was the only reason for failure in our current study, and no case of failure due to instability or loosening was observed. However, the longer average follow-up period in the previous study (7.2 years) than in our present study (4.1 years) could contribute to this difference between them.

There are some data about the KSKS and KSFS results after revision TKA with the use of a PS or a constrained condylar prosthesis. In the current study, we did not compare this prosthesis to other constrained condylar knee prostheses. In addition, we did not compare this prosthesis to other type of prosthesis (PS prosthesis and linked-hinge prosthesis). Studies comparing the outcome of revision TKA using these prosthesis are needed for the future. Haas et al.^15^ reported that because constrained condylar prostheses were used in knees with greater soft-tissue damage, the knee scores were slightly higher for knees with PS prostheses than for those with constrained condylar knee prostheses. Some studies have reported that KSKS and KSFS are markedly improved after revision TKA using a constrained condylar knee prosthesis, as reflected by an increased walking distance and decreased pain.^2^ ^,^ ^6^ Peters et al.^14^ reported the results after an average of five years of follow-up of 57 revision TKAs; these included 14 cases with PS prostheses, of which three failed because of instability, and 43 with constrained condylar prostheses, of which only one failed (due to aseptic loosening). However, Vince et al.^18^ reported a high rate of aseptic loosening after revision TKA using a constrained condylar prosthesis. They added that most of the patients who experienced failure after revision surgery had a history of infection.

Insofar as we know, only a few studies have reported the outcomes of revision TKA using LCCKs (Table 3). Kim et al.^17^ reported that in 114 cases of revision TKA with an average follow-up of 7.2 years, KSKS and KSFS improved from average values of 35 and 16 points, respectively, before revision surgery to 90 and 64 points, respectively, at the final follow-up examination. Furthermore, the average ROM improved from 95° to 106° and the average femorotibial angle from 180.81° to 174.5° as of the final follow-up examination. Similarly, Lee et al.^16^ reported that in 79 cases of revision TKA followed up for an average of 5.3 years, the average values of KSKS and KSFS improved from 48.3 and 36.9 points, respectively, before revision surgery to 88.8 and 73.4 points, respectively, at the final follow-up examination. Moreover, the average ROM improved from 95.1° to 108.0° and the average femorotibial angle from 178° to 174° at the final follow-up examination. Lastly, Hwang et al.^19^ reported that in 15 cases of revision TKA followed up for an average of 2.4 years, the average values of KSKS and KSFS improved from 27 and 44 points, respectively, before revision surgery to 81 and 85 points, respectively, at the final follow-up examination, while the average ROM improved from 80° to 90° and the average femorotibial angle from 181.8° to 176.9° at the final follow-up examination.

**Table 3:** Summary of the clinical studies about outcomes of revision total knee arthroplasty performed with a Legacy Constrained Condylar Knee prosthesis (LCCK).

	Cases	Follow-up periods (years)	KSKS (mean)	KSFS (mean)	ROM (mean, °)	FTA (mean, °)
Kim YH et al.^17^	114	7.2	90	64	106	174.5
Lee JK et al.^16^	79	5.3	88.8	73.4	108.0	174.0
Hwang SC et al.^19^	15	2.4	81	85	90	176.9
Current study	41	4.1	82.9	79.2	105.6	174.9

KSKS: Knee Society knee score; KSFS: Knee Society function score; ROM: range of motion; FTA: femorotibial angle.

Although the complication rate in this current series of revision TKAs using the LCCK was higher than that reported for primary TKA,^7^ ^,^ ^8^ all complications were treated successfully by re-revision with an LCCK or insert exchange alone. From this experience, we anticipate that most complications after revision TKA using the LCCK could also be managed by re-revision with an LCCK. We believe that this relatively new prosthesis could be a useful therapeutic option for patients who need revision surgery.

This study had several limitations. This was a retrospective review of a case series. We did not perform a randomized cohort study comparing this prosthesis to other constrained condylar knee prostheses. The mean follow-up period of 49.4 months (range, 24 to 95 months) was relatively short, and the results might change after longer follow-up. This was not a single-surgeon cohort study, although the techniques used for ligament balancing and fixation were almost the same. Lastly, we did not record any patient-derived outcome scores such as the Knee Society's new scoring system, the New Knee Society Knee Scoring System (New KSKSS).^20^

## CONCLUSION

This study showed that the use of the second-generation constrained condylar knee prosthesis in revision TKA produced excellent clinical and radiographic results. Reports about the outcomes when other types of constrained condylar knee prosthesis are used are needed to judge whether this joint prosthesis is advantageous for patients who undergo revision TKA.
